# Mapping research trends regarding the mechanism of dysphagia from 1993 to 2023: a bibliometrics study and visualization analysis

**DOI:** 10.3389/fneur.2024.1363928

**Published:** 2024-07-04

**Authors:** Qiuping Ye, Jiahui Hu, Yong Dai, Hongmei Wen, Zulin Dou

**Affiliations:** ^1^Department of Rehabilitation Medicine, The Third Affiliated Hospital of Sun Yat-sen University, Guangzhou, China; ^2^Clinical Medical College of Acupuncture Moxibustion and Rehabilitation, Guangzhou University of Traditional Chinese Medicine, Guangzhou, China

**Keywords:** dysphagia, mechanism, CiteSpace, visualization analysis, research frontiers, emerging trends

## Abstract

As a common consequence of various neurogenic disorders, dysphagia has a significant impact on the quality of life for patients. To promote the development the field of swallowing, it will be helpful to clarify the pathological and therapeutic mechanisms of dysphagia. Through visual analysis of related papers from 1993 to 2023 in the Web of Science Core Collection (WoSCC) database, the research status and development trend of the pathogenesis of dysphagia were discussed. The co-occurrence study was finished using CiteSpace 6.2 R4 software, including keywords, countries, institutions, and authors. Finally, 1,184 studies satisfied the inclusion requirements. The findings of the visualization analysis suggested that aspiration and gastroesophageal reflux disease would be the areas of greatest interest for researchers studying the mechanism of dysphagia. As for the latest occurred research trends, fMRI, signals and machine learning emerging into the field of view of researchers. Based on an analysis of country co-occurrence, United States, Japan and China rank the top three, in terms of the number of publications on dysphagia. University System of Ohio is the organization that has published the most amount of articles regarding the mechanism of dysphagia. Other highly published schools in the top three include State University System of Florida and Northwestern University. For the prolific authors, German, Rebecca Z published the most articles at present, whose own research team working closely together. Several closely cooperating research teams have been formed at present, including the teams centered around German, Rebecca Z, Warnecke, Tobias and Hamdy Shaheen. This study intuitively analyzed the current research status of the mechanism of dysphagia, provided researchers with research hotspots in this field.

## Introduction

Dysphagia is a clinical manifestation in which the structure or function of the mandible, lips, tongue, soft palate, throat, esophagus and other organs are impaired, leading to the food cannot be safely and effectively transported from the mouth to the stomach (1). Inability to eat, delayed swallowing, or aspiration of food are characteristics of dysphagia. Dysphagia occurs in up to 81% of patients after stroke or 80% of Parkinson's disease (PD) (2, 3), and can also occur in other neurogenic diseases (4–6), such as head and neck tumor surgery (7). According to research, dysphagia also occurs in the normal people over the age of 60, with a prevalence of about 40% (8).

However, the incidence of dysphagia is influenced by the means of assessment and may be greater depending on the method, timing and criteria used to diagnose dysphagia. In the acute phase, gold-standard routine tests such as VFFS and FEES have practical limitations in that they can only detect obvious dysphagia and therefore miss silent inhalations (9). Therefore, various of measurement have been carried out to correctly diagnose dysphagia, such as bedside evaluation, which can improve the diagnosis of aspiration in the elderly, with high sensitivity in acute stroke patients (10, 11). At the same time, dysphagia after stroke is also affected by a variety of factors, such as NIHSS value, cognitive dysfunction, and the degree of white matter disease, which were described as independent predictors of post-stroke dysphagia and persistent dysphagia at 14 days. So was the pneumonia post-stroke (12, 13).

Dysphagia can lead to a variety of adverse complications, for instance, aspiration, malnutrition, dehydration, and asphyxia (14). It can even lead to serious consequences, such as aspiration pneumonia or death from choking on large food pellets. For those complications, aspiration is easily overlooked, yet many studies have focused on dysphagia without considering aspiration (15). Therefore, highly sensitive dysphagia bedside screening tests (BSEs) designed to detect aspiration and tested against FEES are more likely to describe the real incidence rate (13, 16–18). However, most dysphagia screening often fail to detect silent aspiration, which can cause most dysphagia associated pneumonia (13). Specifically, diagnosing dysphagia after stroke by focusing on obvious signs of aspiration, such as coughing or voice changes, can lead to silent aspiration going undiagnosed, increasing the relative risk of pneumonia and resulting in a poor clinical prognosis due to false negative results (13). Therefore, more clinical attention to screening aspiration, especially silent aspiration, are particularly important. Actually, the combination of BSEs and other methods can improve the detection rate and avoid the misdiagnosis of silent aspiration. As far as we know, there has been research on the detection of silent aspiration, and the project is currently in progress (19). Due to the complexity of its etiology and serious consequences, dysphagia attracted the attention of researchers, and more and more studies on the mechanism of dysphagia are emerging.

In recent years, studies focused on the mechanism of dysphagia have been conducted from the perspectives of central and peripheral. With the in-depth research and increasing interest in the field of swallowing disorders, studies focused on the current situation and trends in this field are needed. After reviewing the literature, we found four related visualization studies that payed attention to the dysphagia (20), post-stoke dysphagia (PSD) (21, 22) or rehabilitation of PSD (23). Those studies analyzed the current literature or the rehabilitation on dysphagia or PSD to identify the research hotspots and frontiers using bibliometrics. However, until now, scientometric analysis in the mechanism of dysphagia has rarely been reported. CiteSpace is one of the most widely used visualization tools, as a large amount of data have been transformed into more intuitive visual maps that can reveal the frontiers and trends in certain fields. It is an influential software in the field of information mapping and visualization that can display the knowledge domain of related disciplines within a certain period of time through intuitive visualization analysis (24). CiteSpace intuitively highlights the quantitative features of research articles in the field, helping researchers understand the evolution of certain field over time (25).

In our study, we used CiteSpace software to uncover cooperative networks and evaluate the research frontiers and emerging trends in the mechanism of dysphagia, and a visual map of keywords, countries, institutions, and authors was generated. Therefore, we will comprehensively and objectively integrate and elaborate the core and frontier information in the field of dysphagia to provide objective and visualization data for relevant researchers.

## Methods

### Data sources

**Figure 1:**
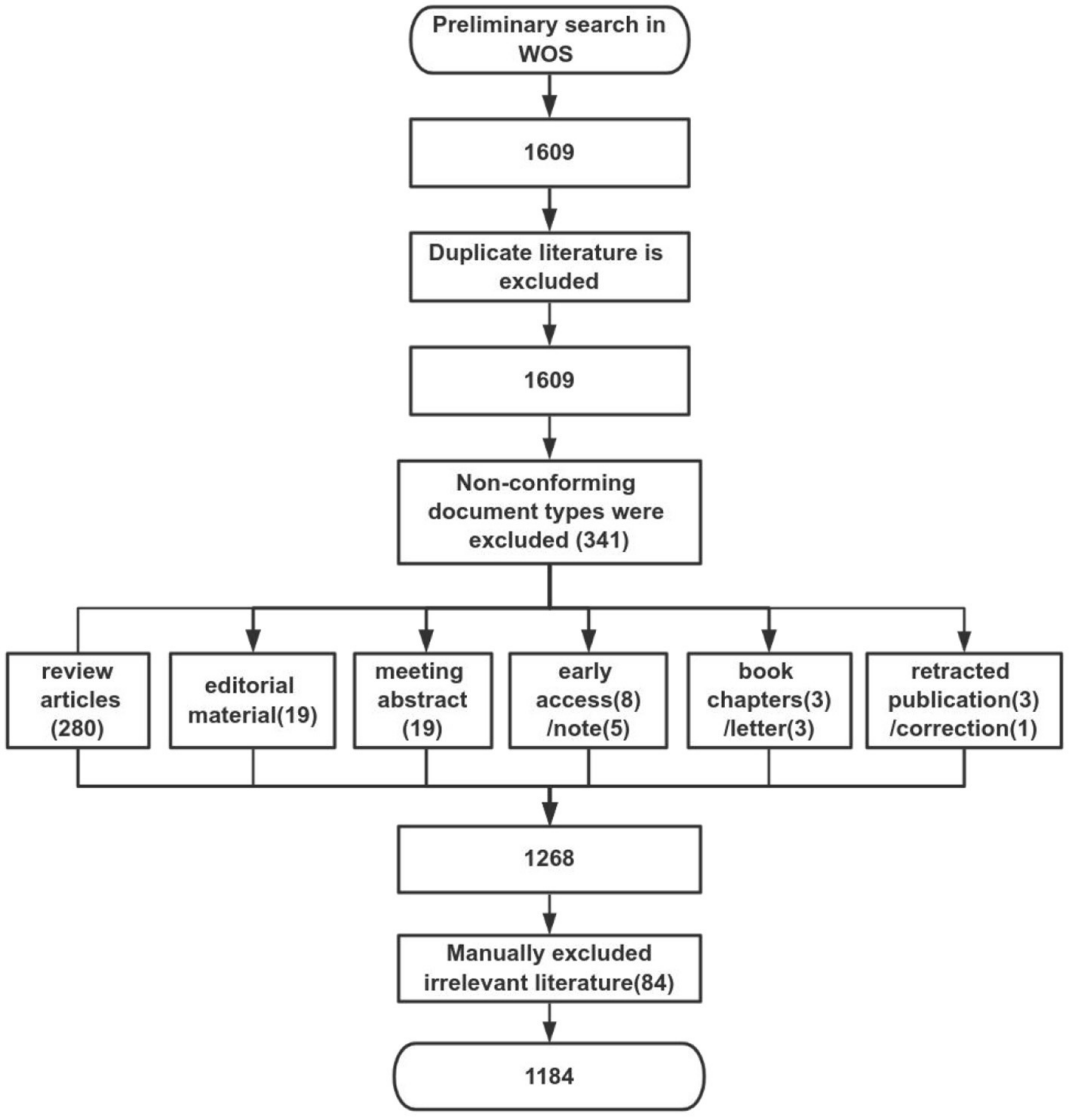
The study tree of the whole research.

Studies retrieval: the search terms for the topics of study were “dysphagia” or “swallowing dysfunction” or “swallowing disorders” and “mechanism”, which were searched from the Web of Science Core Collection (WoSCC) database. The search period was from January 1, 1993, to November 30, 2023. Selection: studies on humans or animals of mechanism on dysphagia were both included. The research type is defined as original research. Exclusion: review articles, editorial material, meeting abstract, early access, note, book chapters, letter, retracted publication and correction. The research process was as follows (Figure 1): a total of 1,609 relevant studies were initially retrieved, 341 documents that did not fit the type were excluded. And 84 irrelevant documents were excluded through examination and abstract reading. As a result, 1,184 papers were finally included.

### Data processing

The raw data from WoSCC were searched and downloaded, and the title and abstract were screened by two independent reviewers (Qiuping Ye and Jiahui Hu). The articles included in the study were exported from the WoSCC database in plain text format and named separately in the download_xxx.txt way. The exported information of the articles included title, year of publication, author, research institution, keywords, abstract, journal, etc. CiteSpace 6.2. R4 software (Drexel University, Philadelphia, PA, USA) was used to process and transform the data.

### Statistical methods

All data were analyzed in CiteSpace 6.2. R4 software, and the node type included the author, institution, and time span. The time span of the keyword node was set to 3 year. The selection criteria were set to the g index *k* = 25, the top 50% level of the most cited or occurred items from each slice. Statistical analysis of keyword co-occurrence analysis, keyword clustering, country and institution co-occurrence analysis, and author co-occurrence analysis, were all processed by CiteSpace. The concrete principles of these methods refer to previous studies (26), which indicated three types of analysis: network visualization, overlay visualization, and density visualization. The nodes in each knowledge graph represented different publications, and the size of the nodes was proportional to their frequency of occurrence in a specific period. Similarly, the connection between nodes indicates the degree of relationship; the thicker the lines are, the stronger the connections. These analyses will make the study field more intuitive, allowing people to observe the trends of various research hotspots over time (27).

## Results

**Figure 2:**
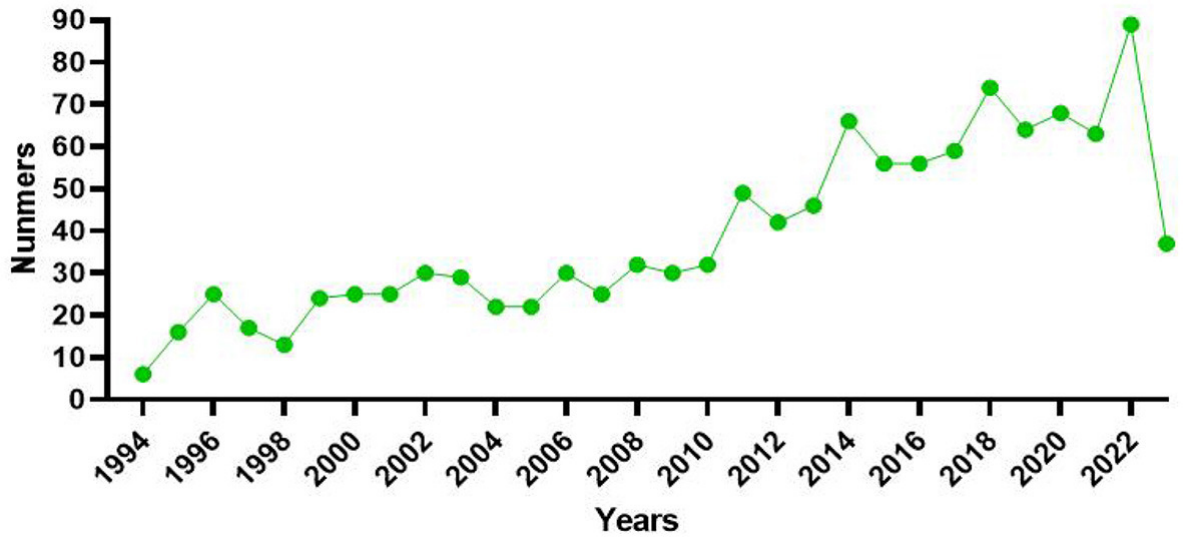
The number of publications from 1993 to 2023 is described by a line chart. The green points indicated the numbers of publications.

To some certain extent, the development level and research popularity of the field can be inferred from the statistics on the number of published papers each year. From 1993 to 2023, a total of 1,184 literature were published. This field saw modest growth from 1994 to 2022 and a downward trend in 2023 as a result of incompletely obtained data from 2023. Up until the point of the literature search, the number of publications reached its peak in 2022 with 89 citations. Figure 2 depicted a stable rise during the entire development phase.

### Co-occurrence analysis of keywords

**Figure 3:**
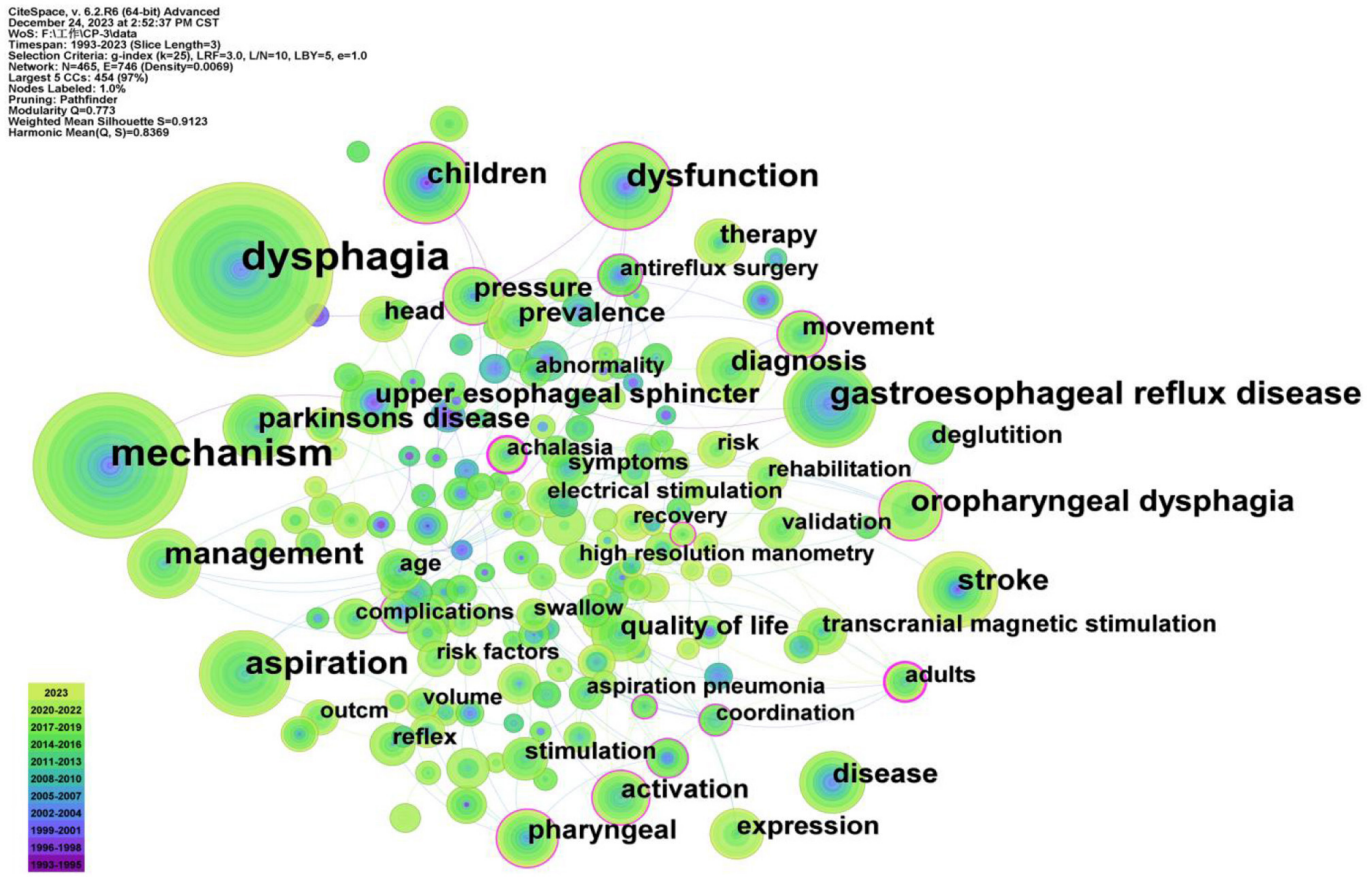
Co-occurrence analysis of the keywords. The thickness of the line indicates the strength of the connection between nodes. The node size indicates the frequency of keywords. In the figure, gastroesophageal reflux disease and aspiration occupied the main position except for dysphagia and mechanism.

**Figure 4:**
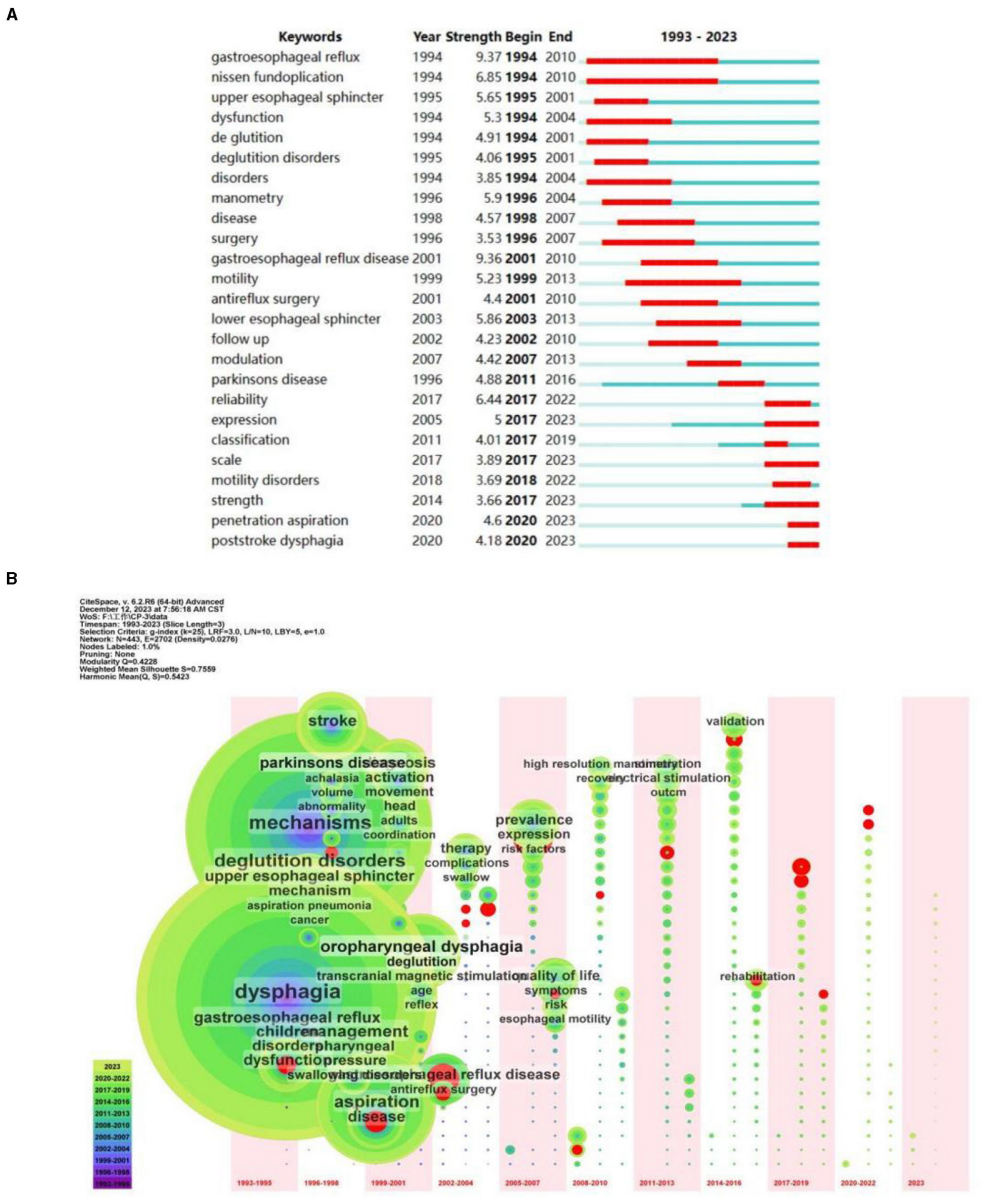
**(A)** The top 25 keywords with the strongest citation bursts. **(B)** The time zone of keyword clustering.

Keywords are a high summary of the core content of the article, and the research hotspots in this field can be judged by the co-occurrence analysis of keywords. In this study, the top 50 most-cited or -occurring items were chosen from each slice. Following the co-occurrence of keywords, 465 nodes and 746 lines were ultimately produced; more than 14 keywords appeared more than 50 times, with the top 10 appearing in Table 1. As illustrated in Figure 3, nodes stand in for keywords, and each node's size reflects how frequently the keywords occur together. There was a strong correlation between the keywords in this article, as evidenced by the line that shows the strength of the link between them. When paired with Table 1, the highest frequency was 108 for “gastroesophageal reflux disease,” 100 for “aspiration,” and “dysfunction,” with the exception of dysphagia (362) and mechanism (249). As for the latest keywords, fMRI, signals and machine learning occurred more frequent (Table 2), which indicating more new research techniques and directions emerged in the mechanism of dysphagia. It is worth noting that, dysphagia caused by interior cervical discectomy is a new vision for researchers, although only once, but in reminding our researchers need to pay attention to dysphagia caused by various diseases. The colored nodes, which represented the keywords, showed the growing trends in the data; the larger the node, the more frequent it is, which indicated the emerging trends in the field of dysphagia. The results indicate a substantial correlation with other terms, with adults having the strongest betweeness centrality (0.23) among all the categories. Figure 4A displayed the top 25 strongest citation bursts. Citation bursts for gastroesophageal reflux disease was the strongest; they began in 1999 and ended in 2010. The red dots in Figure 4B that were obtained based on the results of Figure 3 also highlighted the recent emergence of penetration aspiration and post-stroke dysphagia in 2020. The historical progression of dysphagia research is depicted in Figure 4B, and the citation bursts in Figure 4A are indicated by the red nodes in Figure 4B.

**Table 1:** The top 10 keywords in the co-occurrence analysis.

**Rank**	**Count**	**Centrality**	**Keywords**
1	362	0.04	Dysphagia
2	249	0.05	Mechanism
3	108	0.07	Gastroesophageal reflux disease
4	100	0.05	Aspiration
5	100	0.12	Dysfunction
6	77	0.08	Management
7	67	0.11	Oropharyngeal dysphagia
8	63	0.15	Children
9	60	0.05	Stroke
10	52	0.02	Parkinson's disease

**Table 2:** The latest keywords in the co-occurrence analysis.

**Rank**	**Count**	**Centrality**	**Keywords**
1	3	0.04	fMRI
2	2	0.03	Signals
3	2	0.02	Machine learning
4	2	0.02	MRI
5	1	0.02	Anterior cervical diskectomy

### Cluster analysis of keywords

**Figure 5:**
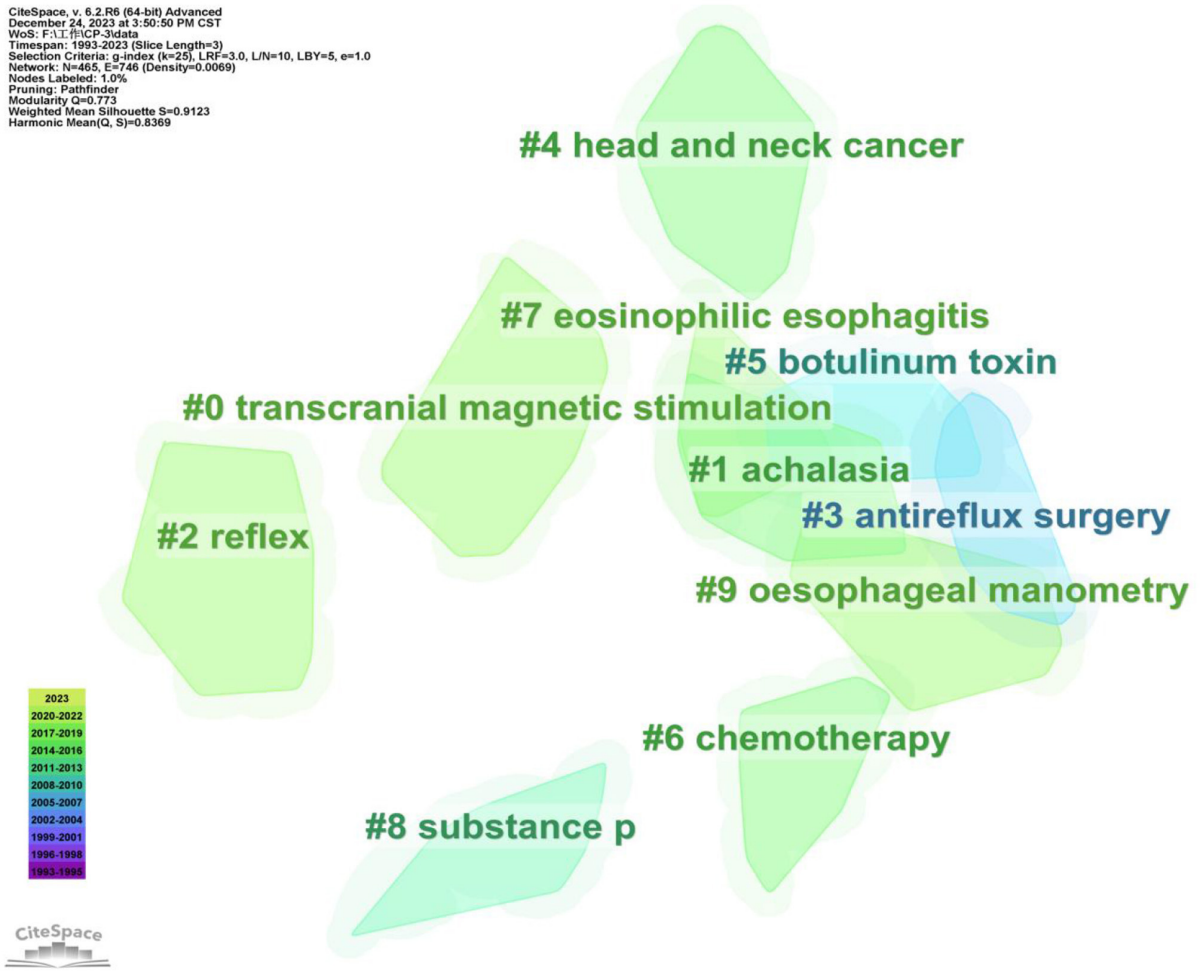
Cluster analysis of the keywords. In clustering, the same elements are aggregated together, each cluster exists independently, and the cluster label is a name for each cluster diagram. The top 10 larger cluster labels were retained in the figure. Different colors display different clusters. The largest three clusters are cluster 0, 1, and 2.

Cluster analysis of keywords can reflect research hotspots and research frontiers in this field. We finally obtained 20 clustering tags, and the value of the keywords cluster module was (Q) = 0.773 > 0.3, indicating that this cluster structure was significant (Figure 5). The average silhouette was (S) = 0.9123 > 0.7, indicating that the members of the clusters had good homogeneity and high confidence. The first 10 cluster labels were retained in this study, including transcranial magnetic stimulation (TMS), achalasia, reflex, antireflux surgery, head and neck cancer, botulinum toxin, chemotherapy, eosinophilic esophagitis, substance p, and esophageal manometry. These groupings comprised molecular mechanisms, diseases, therapeutic strategies, and evaluation methodologies. Based on the included literature, we could determine that dysphagia caused by stomach and esophagus problems concerned the researchers. As for types of disease, researchers mainly focused on dysphagia after head and neck cancer except for stroke. Among those clusters, TMS occurred in a relatively new time in 2014, which indicated the research trends and hot spots for the treatment of dysphagia.

### Distribution of countries and institutions

**Figure 6:**
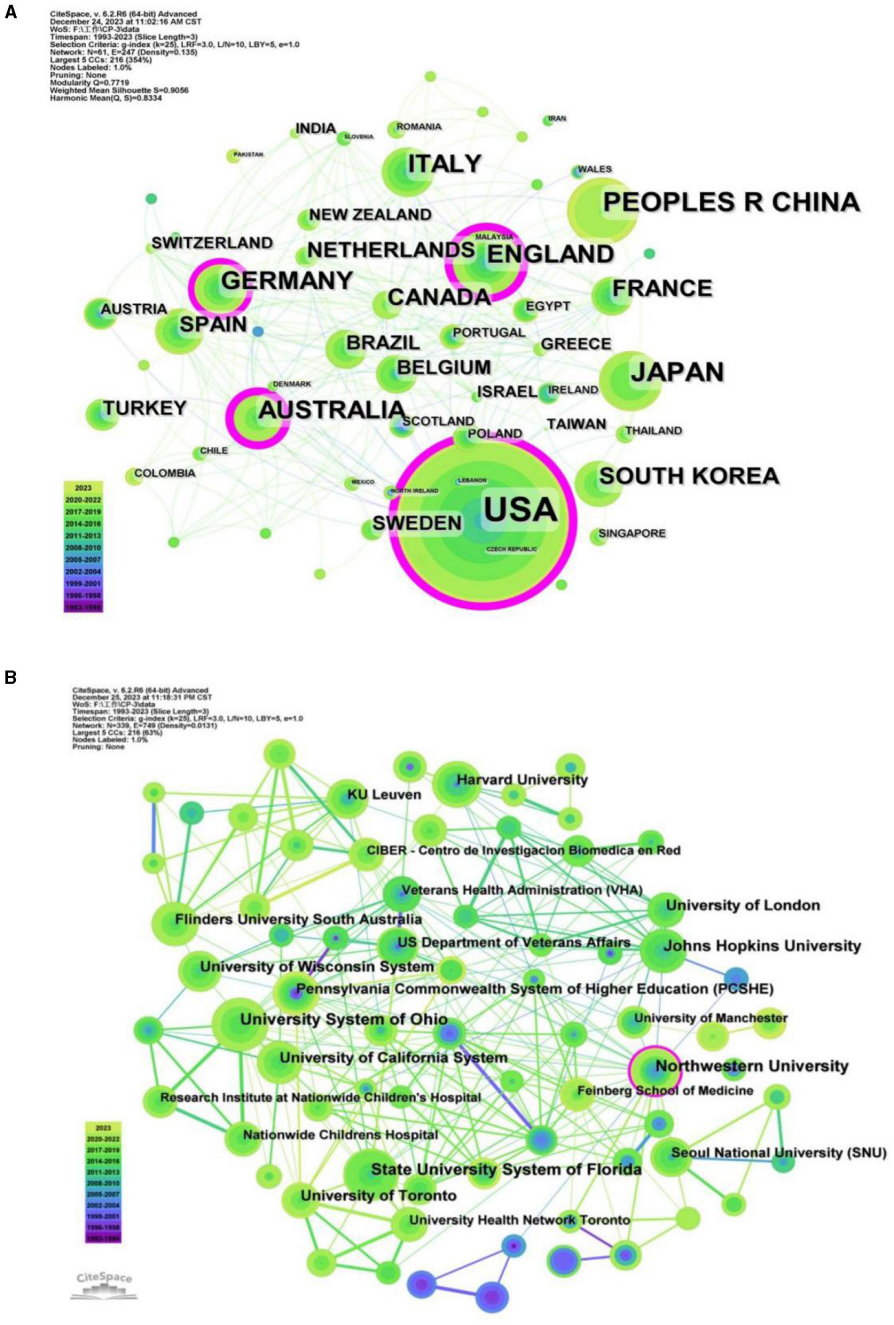
**(A)** Co-occurrence analysis of the countries. **(B)** Co-occurrence analysis of the institutions.

We were able to identify countries and institutions that have shown a greater interest in the mechanism of dysphagia and have made significant contributions to the field's progress through the examination of published literature. The co-occurrence analysis of countries and institutions reflects the distribution of major scientific research forces in this field. 61 nodes and 247 links were produced by the co-occurrence analysis atlas of the countries, of them, nine countries produced more than 50 articles (Table 3). The United States, Japan, and China are the top three contributors, accounting for 43% of all literature. Figure 6A depicted the international collaboration network, with link strength indicating the degree of cooperation between countries. USA was the most prolific country with 410 articles, followed by the Japan (116 papers) and China (92 articles). The most three contributing countries, however, have weak links and limited mutual cooperation, although they have all developed close relationships with other countries. Therefore, in order to facilitate the sharing of beneficial resources and the advancement of the field, the major contributing nations should step up their cooperation.

**Table 3:** The top 9 countries that published more than 50 articles in the co-occurrence analysis.

**Rank**	**Count**	**Centrality**	**Countries**
1	410	0.39	USA
2	116	0.02	Japan
3	92	0.02	People's Republic of China
4	79	0.34	England
5	70	0.07	Italy
6	66	0.24	Australia
7	66	0.12	Germany
8	54	0.1	France
9	54	0	South Korea

With 339 institutions, the co-occurrence analysis atlas of institutions produced 339 nodes and 749 lines, showing a comparatively large number of research groups, the majority of which collaborated closely with each another. University System of Ohio, State University System of Florida, and Northwestern University were the top three significant output institutions, with respective outputs of 33, 31, and 31 (Figure 6B). However, 70% of the top 10 organizations came from the United States (Table 4), indicating an imbalanced distribution of research on the mechanism of dysphagia around the world. The three institutions with the most published papers are relatively independent in their research, which need more cooperation and exchange to promote the development of the industry.

**Table 4:** The top 10 institutions in the co-occurrence analysis.

**Rank**	**Counts**	**Centrality**	**Institutions**
1	33	0.06	University System of Ohio
2	31	0.02	State University System of Florida
3	31	0.27	Northwestern University
4	25	0.04	Johns Hopkins University
5	24	0.05	University of California System
6	24	0.02	University of Wisconsin System
7	22	0.01	University of Toronto
8	22	0.07	University of London
9	20	0.07	Harvard University
10	19	0.03	Flinders University South Australia

### Analysis of authors

**Figure 7:**
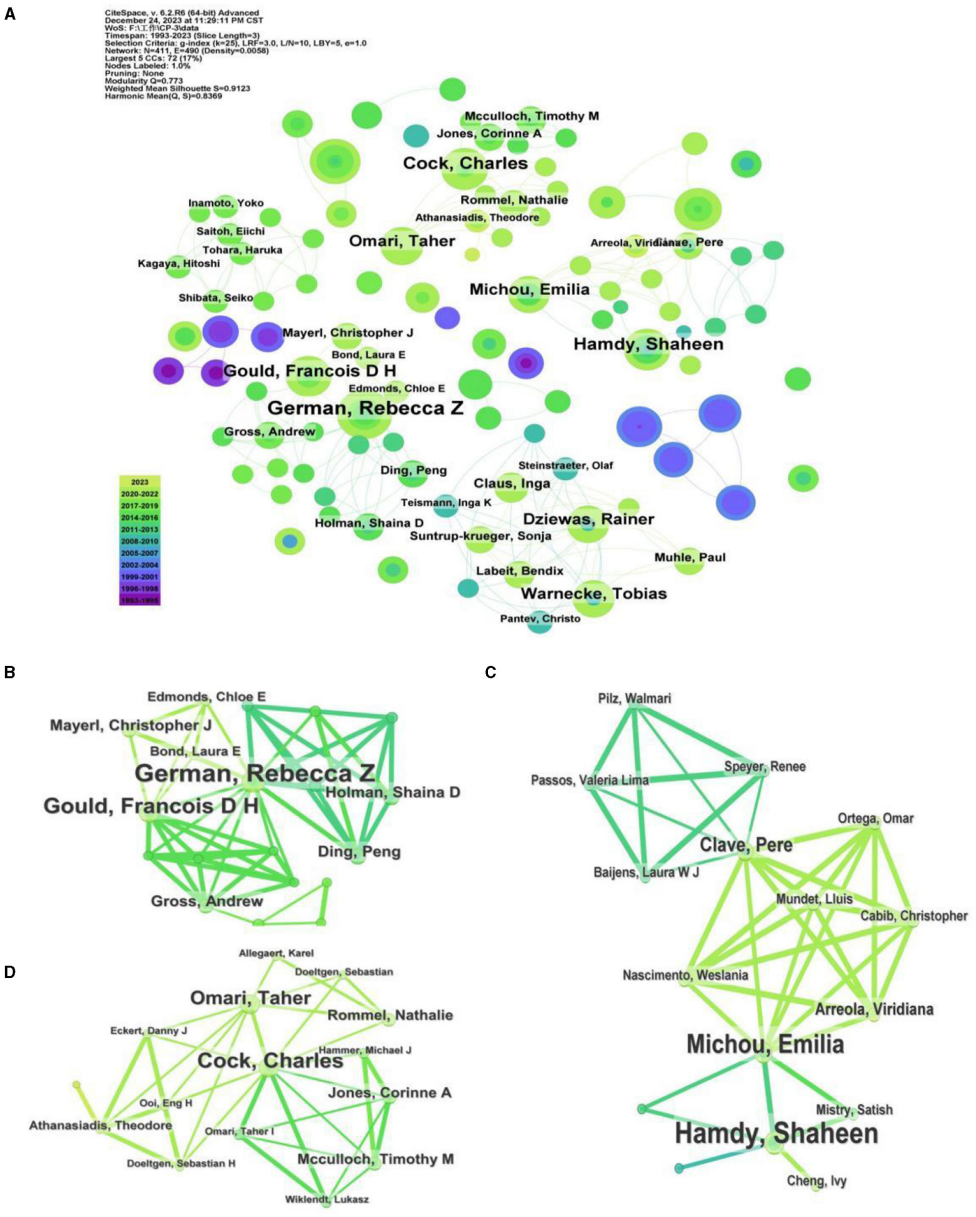
**(A)** Co-occurrence analysis of the authors. **(B–D)** Co-occurrence analysis of the closely work authors.

The co-occurrence analysis of authors can reflect the cooperation and communication between academic leaders and authors in the field. In this study, the co-occurrence analysis of authors generated 411 nodes and 490 lines (Figure 7A). The top-ranked authors who have most publications were German, Rebecca Z with 14 documents (Table 5). The most citation documents of German, Rebecca Z was an article published in the journal of Dysphagia with 31 citation, which studied the relationship of unilateral superior laryngeal nerve (SLN) lesion and dysphagia and aspiration (28). However, his article published in the relative lower impact factor (IF) journal, among which 4 article published in the journal of Dysphagia (28–31) and 2 published in the journal of LARYNGOSCOPE (32, 33), both of which have the IF of 2.6. Dysphagia was an influential journal in swallowing and dysphagia field that has 5,546 total citations in 2022, while 28,923 total citations were seen in the journal of LARYNGOSCOPE. Authors who have second-ranked publications was Jadcherla, Sudarshan R with 12 documents, following with Cock Charles, Steele Catriona M and Hamdy S, all of them has published 10 documents. On the top 10 authors who have most publications, Aydogdu I has most total citations with 625 and average citation with 69, following with Ertekin C with 334 total citations and 42 average citations. The highest IF journal they published is the journal of Brain with the IF of 14.5 in 2022, following with the journal of Neurology Neurosurgery and Psychiatry with the IF of 11.1. Furthermore, the average IF they published reached up to 6.82. Those data showed that they are the influential authors in this field. Interesting, they belong to the same team, which indicated that it is a team with close cooperation, rich results and high impact.

**Table 5:** The top 10 authors in the co-occurrence analysis.

**Rank**	**Author**	**Documents**	**Citations**	**Average citation**
1	German, Rebecca Z	14	198	17
2	Jadcherla, Sudarshan R	12	146	13
3	Cock, Charles	10	105	11
4	Steele, Catriona M	10	215	23
5	Hamdy, Shaheen	10	284	36
6	Gould, Francois D H	9	108	15
7	Aydogdu, I	9	625	69
8	Michou, Emilia	8	279	62
9	Warnecke, Tobias	8	159	23
10	Ertekin, C	8	334	42

In additionally, there is little collaboration between the three most prolific authors, and they have their own independent research collaboration. Previous study showed that the top three authors with the most publications were Dziewas R, Hamdy S and Clave P (21), but two of them have published fewer than 10 research articles on the mechanisms of dysphagia. Similarly, the three teams with the largest volume of publications were decentralized and did not collaborate with each other. It is worth noting that the team centered around German, Rebecca Z and Gould, Francois D H formed a tight network model; Another tight collaboration team centered on Warnecke T and Dziewas R, Hamdy S and Michou E, Cock C and Omari, T (Figures 7B–D), among which, the team of Hamdy S and Michou E has high total and average citations. These figures reveal several stable groups of teamwork have been formed, including a collaborative research group centered on German Rebecca Z, Warnecke T, Hamdy S.

## Discussions

Dysphagia is a prevalent complication among neurogenic diseases, which seriously affect both the life of patients and the burden of family and society. Deeply analyzing the pathogenesis of diseases can help people take more effective preventive measures, which need mechanism researches. In this paper, relevant literature on the mechanism of dysphagia in WoSCC database in recent 30 years was used as the research object. They were visually analyzed by extracting the information about keywords, authors, countries, institutions. In the form of knowledge graph, we analyzed the current research status about mechanism of dysphagia from different perspectives, and discussed the research frontiers and trends in this field.

In the historical process of the mechanism of dysphagia, the study of central mechanism is the earliest and most studied, especially in stroke (34). We could also obtain that among diseases, stroke and Parkinson's disease appeared in the top 10 co-occurrence keywords, and they also have relative strongest citation bursts (Figure 4A, Table 1). For dysphagia after stroke, mechanism focused mostly on swallowing primary motor cortex (35) and excitatory neurons in swallowing related brains, including cortex and subcortical brain areas (34, 36, 37). Recently, the cerebellum has been considered as another regulatory brain region in recovery of dysphagia and may play an important role in bidirectional regulation (38). It is worth noting that the cortical compensation mechanism, such as the pharyngeal representations of unaffected hemisphere are important, which have been proposed in Gastroenterology in 1998 (39). This direction is still being investigated in the treatment of dysphagia until now. With the development of science and technology, neural circuits have attracted the attention of researchers (40), so various neuroscience methods, including optogenetics, chemogenetics and two-photon imaging, have been applied in this process.

For the latest keywords in the co-occurrence analysis of dysphagia, we could see that some emerging vision come into our view, including fMRI, signals and machine learning (Table 2). In the signal transmission of swallowing brains, excitatory neurotransmitters have received the most attention (34), while gamma aminobutyric acids (GABAs) is another neurotransmitter that can inhibit the activation of swallowing (41) and it also involved in suppressing the swallowing reflex after harmful irritation of the facial and oral structures (42). However, there has been little research in recent years. Furthermore, increases in exercise-related BDNF and tyrosine kinase receptor B (TrkB) may play a role in the mechanism that promotes increased tongue strength in young and middle-aged rats (43). In a previous study, 5-HT in the NTS also played a critical role in EA treatment of swallowing (44). In PD, the recovery of dysphagia may be associated with reduced presynaptic dopaminergic integrity in the caudate nucleus (45), but other researchers have not endorsed this idea (46). In recent years, gene regulation has attracted the attention of researchers (47), which needs in-depth study. The researchers proposed that the cerebral intestinal axis (48) and genes, such as polymorphisms of the brain-derived neurotrophic factor (BDNF) gene (49, 50), are associated with the recovery of dysphagia. In general, there are more and more studies on the mechanisms of swallowing, providing more scientific basis for the revealing the mechanism of dysphagia.

The results of cluster analysis of the keywords showed that TMS occupied an important category. As a non-invasive brain stimulation for the rehabilitation of dysphagia, TMS, especially repetitive TMS (rTMS) was mostly used. The group that has studied rTMS the most is Hamdy S 'team, whose research focuses mostly on the human pharyngeal motor cortex (51, 52). Those studies uncovered the underlying mechanism of rTMS treatment referring to the excitatory of cortex and cortical plasticity. Furthermore, except for the cortex, rTMS also regulates the cerebellum, which also increased the excitatory of pharyngeal motor cortex (53). However, the same stimulation on cerebellar vermis with rTMS took an opposite inhibitory effect to pharyngeal motor cortical activity and swallowing behavior (54). The effect of cerebellar on dysphagia attracted the attention of more and more researchers in recent year, which is a research frontier of the underlying mechanism of dysphagia. Researchers thought that TMS is a neurostimulatory techniques hold future therapeutic promise.

## Limitations

Our study had some limitations. First, we only concentrated on visualization analysis of the mechanism of dysphagia, but did not distinguish and mention the mechanisms of specific treatment methods for dysphagia. Second, since the database we searched was only WoSCC database, the literature included was not comprehensive. More in-depth and comprehensive studies should be carried out in the future.

## Conclusions

Research on aspiration concerned the researchers and practitioners, indicating the important of safety in dysphagia. As for the latest occurred research trends, fMRI, signals and machine learning emerging into researchers' eyesight. This results showed that collaboration between nations, institutions and regions is uncommon and has to be enhanced going forward. Here, we propose that increased collaboration between specialists from many fields and nations is necessary to implement high-quality multicenter research.

The study offers insights into the mechanism behind dysphagia from the perspective of visualization approach, which could assist researchers and practitioners in comprehending more comprehensive of dysphagia.

## Author contributions

QY: Conceptualization, Funding acquisition, Software, Writing – original draft, Formal analysis. JH: Data curation, Investigation, Methodology, Writing – review & editing. YD: Data curation, Project administration, Validation, Writing – review & editing. HW: Supervision, Visualization, Writing – review & editing. ZD: Resources, Visualization, Writing – review & editing, Supervision.
